# N4‐Acetylcytidine Drives Glycolysis Addiction in Gastric Cancer via NAT10/SEPT9/HIF‐1*α* Positive Feedback Loop

**DOI:** 10.1002/advs.202300898

**Published:** 2023-06-16

**Authors:** Qingbin Yang, Xuetao Lei, Jiayong He, Yanmei Peng, Yihao Zhang, Ruoyu Ling, Chaorui Wu, Guofan Zhang, Boyang Zheng, Xinhua Chen, Boya Zou, Ziyi Fu, Liying Zhao, Hao Liu, Yanfeng Hu, Jiang Yu, Fengping Li, Gengtai Ye, Guoxin Li

**Affiliations:** ^1^ Department of General Surgery Nanfang Hospital Southern Medical University Guangdong Provincial Engineering Technology Research Center of Minimally Invasive Surgery Guangzhou Guangdong 510515 P. R. China; ^2^ Guangdong Provincial Key Laboratory of Precision Medicine for Gastrointestinal Tumor Guangzhou Guangdong 510515 P. R. China

**Keywords:** ac4C, gastric cancer, glucose metabolism reprogramming, hypoxias, NAT10

## Abstract

Anti‐angiogenic therapy has long been considered a promising strategy for solid cancers. Intrinsic resistance to hypoxia is a major cause for the failure of anti‐angiogenic therapy, but the underlying mechanism remains unclear. Here, it is revealed that N4‐acetylcytidine (ac4C), a newly identified mRNA modification, enhances hypoxia tolerance in gastric cancer (GC) cells by promoting glycolysis addiction. Specifically, acetyltransferase NAT10 transcription is regulated by HIF‐1*α*, a key transcription factor of the cellular response to hypoxia. Further, acRIP‐sequencing, Ribosome profiling sequencing, RNA‐sequencing, and functional studies confirm that NAT10 in turn activates the HIF‐1 pathway and subsequent glucose metabolism reprogramming by mediating SEPT9 mRNA ac4C modification. The formation of the NAT10/SEPT9/HIF‐1*α* positive feedback loop leads to excessive activation of the HIF‐1 pathway and induces glycolysis addiction. Combined anti‐angiogenesis and ac4C inhibition attenuate hypoxia tolerance and inhibit tumor progression in vivo. This study highlights the critical roles of ac4C in the regulation of glycolysis addiction and proposes a promising strategy to overcome resistance to anti‐angiogenic therapy by combining apatinib with ac4C inhibition.

## Graphical abstract

Intrinsic resistance to hypoxia is a major cause for the failure of anti‐angiogenic therapy. N4‐acetylcytidine (ac4C), a newly identified mRNA modification, is found to enhance hypoxia tolerance in gastric cancer cells by promoting glycolysis addiction. ac4C drives glycolysis addiction via a NAT10/SEPT9/HIF‐1*α* positive feedback loop. Combining ac4C inhibition with apatinib can overcome anti‐angiogenic therapy resistance and optimize anti‐tumor efficacy.

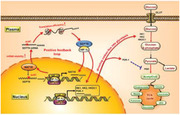

## 1 Introduction

Despite decade‐long advances in gastric cancer (GC) research have significantly improved surgical practices^[^ ^1^, ^2^ ^]^ and comprehensive GC treatment regimens,^[^ ^3^ ^]^ GC remains the fifth most commonly diagnosed cancer and the fourth major cause of cancer‐related deaths worldwide in 2020.^[^ ^4^ ^]^ Anti‐angiogenic treatments (e.g., bevacizumab, apatinib) are well‐established therapies for various tumors, including advanced GC, which improve progression‐free survival (PFS) and overall survival (OS).^[^ ^5^, ^6^, ^7^, ^8^ ^]^ However, anti‐angiogenic therapy also provokes intense hypoxia within tumors.^[^ ^9^, ^10^, ^11^ ^]^ In response to a hypoxic microenvironment, tumor cells undergo glucose metabolism reprogramming for rapid and substantial ATP generation to maintain cell survival and hyperplastic growth, which frequently limits the efficacy of anti‐angiogenic therapies. Therefore, it is essential to unravel the mechanisms of metabolic reprogramming and identify therapeutic targets to disrupt the hypoxia tolerance of tumor cells.

Hypoxia is one of the major features of the tumor microenvironment, triggering cellular glucose metabolism reprogramming.^[^ ^12^, ^13^ ^]^ It means cancer cells rely heavily on glycolysis rather than mitochondrial oxidative phosphorylation (OXPHOS) to sustain the essential biological functions.^[^ ^14^, ^15^ ^]^ As a crucial mediator of the cellular response to hypoxia, the accumulation of HIF‐1*α* activates genes involved in hypoxic homeostatic response, such as hexokinase (HK), lactate dehydrogenase (LDH), and pyruvate dehydrogenase kinase (PDK), to promote glycolysis and inhibit OXPHOS processes.^[^ ^16^, ^17^ ^]^ Thus, overactive HIF‐1 signaling results in glycolysis addiction.^[^ ^18^, ^19^ ^]^ Recently, increasing evidence has revealed that mRNA modifications are widely implicated in the metabolic recombination process.^[^ ^20^ ^]^ For example, N6‐Methyladenosine (m6A) promotes glucose metabolic recombination in cancer cells by activating the mTOR signaling pathway or upregulating the expression of metabolic enzymes such as HK2 and PDK4.^[^ ^20^, ^21^, ^22^ ^]^ Similarly, we observed that N4‐acetylcytidine (ac4C), a newly identified mRNA modification, was remarkably enriched in hypoxic GC cells, which implies a strong link between ac4C and hypoxia tolerance. However, the mechanism by which ac4C regulates hypoxia tolerance in tumor cells remains unknown.

Initially described in tRNAs and 18S rRNA,^[^ ^23^, ^24^, ^25^ ^]^ ac4C was recently discovered to be widespread throughout the human transcriptome.^[^ ^26^ ^]^ As the only known ac4C “writer” protein, NAT10 possesses both acetyltransferase and RNA‐binding activities, enhancing the stability and translation efficiency of mRNA.^[^ ^26^ ^]^ Since then, accumulating evidence indicated that ac4C dysregulation profoundly contributed to the pathogenesis of various diseases, including HIV and enterovirus 71 (EV71) replication,^[^ ^27^, ^28^ ^]^ osteoporosis,^[^ ^29^ ^]^ cardiomyocyte apoptosis,^[^ ^30^ ^]^ and failure of spermatogonial differentiation.^[^ ^31^ ^]^ Moreover, emerging evidence shows that ac4C is strongly associated with tumor progression (e.g., multiple myeloma, pancreatic, bladder, and gastric cancers).^[^ ^32^, ^33^, ^34^, ^35^ ^]^ NAT10 regulates the cell cycle and cancer stemness of bladder cancer cells by mediating mRNA ac4C modification of BCL9L, SOX4, and AKT1.^[^ ^32^ ^]^ In addition, NAT10 promotes cell proliferation by acetylating CEP170 mRNA in multiple myeloma.^[^ ^33^ ^]^ Although Xun Li et al. revealed in 2021 that NAT10 promoted GC cells’ epithelial‐to‐mesenchymal transition (EMT) process by directly mediating ac4C modification of COL5A1 mRNA,^[^ ^35^ ^]^ the functional role of ac4C in GC has not been sufficiently described.

In the current study, we uncovered the relationship between mRNA ac4C modification and hypoxia, and provided a new insight into the ac4C function in GC. By combining the analyses of acRIP‐sequencing (acRIP‐seq), Ribosome profiling sequencing (Ribo‐seq), and RNA‐sequencing (RNA‐seq), we revealed a novel mechanism by which a NAT10/SEPT9/HIF‐1*α* positive feedback loop promotes glycolysis addiction. Based on this mechanism, our study proposed a promising GC strategy that combines apatinib with ac4C inhibition to overcome anti‐angiogenic therapy resistance and optimize anti‐tumor efficacy.

## 2 Results

### 2.1 A Hypoxic Tumor Microenvironment Contributes to the Upregulation of NAT10 in GC

**Figure 1:**
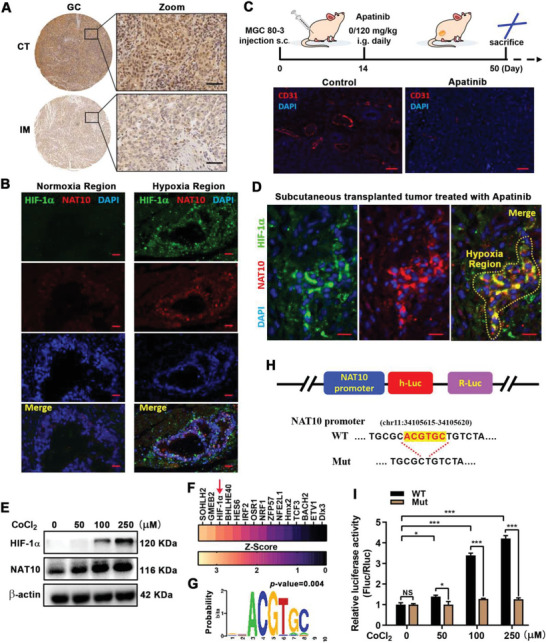
A hypoxic tumor microenvironment contributes to the upregulation of NAT10 in GC. A) The NAT10 expression in CT and IM was detected by IHC. Scale bars, 50 µm. B) Dual staining of HIF‐1*α* and NAT10 to assay the correlation between hypoxia and NAT10 expression in human GC tissues (HIF‐1*α*, green; NAT10, red; DAPI, blue). Scale bars, 20 µm. C) Diagram depicting the construction processes and treatment schedule for the subcutaneous tumor model (upper panel). Tumors treated with apatinib had decreased angiogenesis (lower panel; CD31, red; DAPI, blue). Scale bars, 50 µm. D) The hypoxia and NAT10 expression in tumors treated with apatinib were detected by confocal immunofluorescence microscopy (HIF‐1*α*, green; NAT10, red; DAPI, blue). Scale bars, 20 µm. E) Western blot analysis of NAT10 expression in AGS cells treated with 0–250 µm CoCl_2_. F) Identification of transcription factors interacting with NAT10 promoter by Pscan (http://159.149.160.88/pscan/) online promoter database. G) The motif of HIF‐1*α* binding NAT10 promoter, prompted by Pscan. H) Schematic representation of the mutated promoter in pEZX‐PL01‐NAT10‐luc reporter to investigate the role of HIF‐1*α* in NAT10 expression. I) AGS cells with CoCl_2_ treatment (0–250 µm) were transfected with NAT10 promoter‐WT or Mut reporters for 24 h.

Although previous studies^[^ ^32^, ^35^ ^]^ and our data (Figure S1A‐‐C, Supporting Information) have confirmed that NAT10 expression is upregulated in GC and other tumors, the mechanism responsible for this aberrant expression remains unknown. Intriguingly, immunohistochemistry (IHC) analysis showed that the expression of NAT10 was higher in the center of tumor (CT) than in the invasive margin (IM) (**Figure** **1**A). As is well known, the rapid proliferation of tumor cells and high oxygen consumption frequently result in a hypoxic microenvironment within solid tumors.^[^ ^13^ ^]^ Thus, we further detected the correlation between hypoxia and NAT10 expression in human GC tissues by immunofluorescence (IF) and found that NAT10 was highly enriched in hypoxic GC cells (Figure 1B). Apatinib, a selective VEGFR‐2 tyrosine kinase inhibitor used for advanced GC therapy, has been reported to induce intratumor hypoxia.^[^ ^5^, ^9^ ^]^ Therefore, we treated the subcutaneous tumor model with 120 mg kg^−1^ apatinib (oral gavage, daily), followed by a significant reduction in tumor angiogenesis (Figure 1C). As predicted, pronounced HIF‐1*α* accumulation was observed within the apatinib‐treated tumors, and GC cells within hypoxic regions exhibited enhanced NAT10 expression compared to adjacent non‐hypoxic cells (Figure 1D). In addition, we treated the GC cells with the hypoxia mimetic cobalt chloride (CoCl_2_) to simulate hypoxic conditions in vitro. Treatment with CoCl_2_ increased intracellular levels of HIF‐1*α* and NAT10 in a dose‐dependent manner (Figure 1E), implying that exacerbated cellular hypoxia promotes NAT10 expression.

Moreover, we further investigated the Pscan website (http://159.149.160.88/pscan/) for the vital transcription factors regulating NAT10 expression. The results suggested that HIF‐1*α* may bind to the ACGTGC sequence within NAT10 promoter (Figure 1F,G), which was consistent with the typical hypoxia response element (HRE) sequence (A/G) CGTG.^[^ ^36^, ^37^ ^]^ As the vital transcription factor of HIF‐1 pathway, HIF‐1*α* was required to translocate into the nucleus and bind to the HRE to initiate gene transcription. Therefore, we then constructed luciferase reporter vectors containing wild type or mutant NAT10 promoters targeting this interaction region (Figure 1H). The dual‐luciferase assay showed that the transcriptional activity of luciferase plasmids containing the wild type NAT10 promoter was more active with increasing CoCl_2_, while the mutant groups failed to induce luciferase activity (Figure 1I). Together, our data indicated that the hypoxic tumor microenvironment contributes to the upregulation of NAT10 in GC and that NAT10 transcription is regulated by HIF‐1*α*.

### 2.2 ac4C Promotes Metabolic Rewiring toward a Glycolytic Phenotype in GC Cells

**Figure 2:**
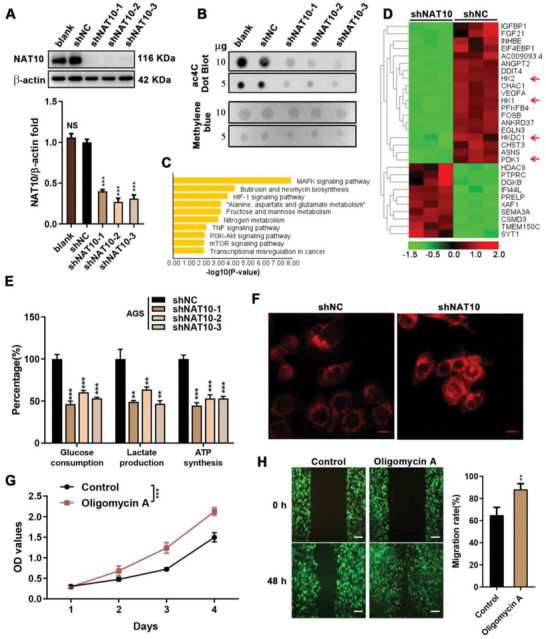
ac4C promotes metabolic rewiring toward a glycolytic phenotype in GC cells. A) Expression of NAT10 in shRNAs transfected AGS cells was analyzed by Western blot and qPCR. B) The total RNA ac4C level in NAT10‐knockdown AGS cells was determined by anti‐ac4C dot blot with methylene blue staining as loading control. C) Representative pathway analysis terms showing pathways of related genes significantly enriched by ac4C. D) Differential genes expression analysis showed that the expression of HK1, HK2, HKDC1, and PDK1 were down‐regulated in shNAT10 cells. E) AGS cells with NAT10 knockdown (treated with 100 µm CoCl_2_) exhibited lower glucose consumption, lactate production, and ATP levels. F) Confocal laser scanning microscope analysis of mitochondrial membrane potential in NAT10‐knockdown AGS cells (treated with 100 µm CoCl_2_) after Mito Tracker Red CMXRos staining. Scale bars, 10 µm. G,H) The proliferation and migration changes of AGS cells treated with Oligomycin A (1 µm) were tested using CCK8 assay G) and monolayer wound healing assay H), respectively. Scale bars, 500 µm.

To investigate the functions of mRNA ac4C modification in GC, we constructed GC cell lines (AGS and MGC 80–3) with stable NAT10 knockdown by transfecting shRNAs (**Figure** **2**A; Figure S2A, Supporting Information). The knockdown of NAT10 expression resulted in a significant downregulation of total RNA ac4C levels (Figure 2B; Figure S2B, Supporting Information). Surprisingly, the RNA‐seq analysis implied that NAT10 regulates the activity of the HIF‐1 pathway in turn. The pathway enrichment analysis and gene set enrichment analysis (GSEA) found that the HIF‐1 pathway and the glycolysis were inhibited in GC cells with NAT10 knockdown (Figure 2C; Figure S2C, Supporting Information). Consistently, differential genes expression analysis and qPCR validation demonstrated that several glucose metabolism key enzymes regulated by the HIF‐1 pathway were down‐regulated in shNAT10 cells, such as Hexokinase (HK1, HK2, and HKDC1) and pyruvate dehydrogenase kinase 1 (PDK1) (Figure 2D; Figure S2D,E, Supporting Information). Hexokinase including HK1, HK2, and HKDC1, is the initial rate‐limiting enzyme of glycolysis, catalyzing the phosphorylation of glucose by ATP to glucose‐6‐P.^[^ ^16^, ^38^, ^39^ ^]^ PDK1 inhibits the conversion of pyruvate into acetyl coenzyme A (acetyl CoA) by phosphorylating pyruvate dehydrogenase (PDH) and ultimately suppresses the OXPHOS process.^[^ ^15^, ^40^, ^41^ ^]^ HK and PDK synergistically direct carbon flux from OXPHOS into glycolysis, driving cellular metabolic reprogramming and glycolysis dependence.

As predicted, the downregulation of metabolic enzymes (HK1, HK2, HKDC1, and PDK1) expression was followed by decreased glycolysis and less mitochondrial damage in hypoxia. The shNAT10 cells treated with CoCl_2_ exhibited significantly lower glucose consumption, lactate production, and ATP levels (Figure 2E; Figure S2F, Supporting Information). The mitochondria in control cells were depolarized along with decreased mitochondrial membrane potential (MMP), reflecting mitochondrial dysfunction (Figure 2F; Figure S2G, Supporting Information). Furthermore, reversing NAT10 expression significantly increased glycolysis while suppressing mitochondrial activity in GC cells (Figure S3A‐‐E, Supporting Information).

To further investigate the effect of metabolic reprogramming on GC cells, Oligomycin A was utilized to enhance cellular glycolysis and hinder the OXPHOS process. The results indicated that increased glycolysis improved the proliferation and migration of GC cells in hypoxia (Figure 2G,H). Likewise, GC cells were more dynamic with higher NAT10 expression (Figure S3F–H, Supporting Information), suggesting that NAT10 can enhance the hypoxia tolerance in GC cells. Collectively, our findings revealed that NAT10 was involved in regulating glucose metabolism reprogramming and enhancing the glycolysis dependence in hypoxia to maintain GC cells' essential biological functions. Additionally, there may be a feedback loop between HIF‐1*α* and NAT10.

### 2.3 SEPT9 Regulates Glucose Metabolism Reprogramming by mRNA ac4C Modification

**Figure 3:**
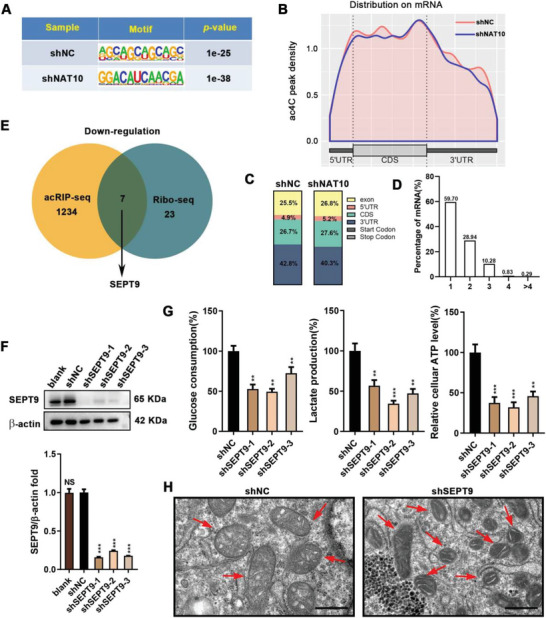
SEPT9 is involved in regulating glycolysis addiction in GC cells. A) Sequence analysis of the highly enriched motif within ac4C peaks in acRIP‐seq. B) Density distribution of ac4C peaks across mRNA transcripts. C) Proportion of ac4C peak distribution in the exon, 5″UTR, start codon, CDS, stop codon or 3″UTR region across the entire set of mRNA transcripts. D) Percentage of mRNAs with different numbers of ac4C peaks. F) Expression of SEPT9 in shRNAs transfected AGS cells were analyzed by Western blot and qPCR. G,H) The GC cells were treated with 100 µm CoCl_2_. G) Reduced glucose consumption, lactate production, and ATP synthesis were observed in SEPT9 knockdown AGS cells. H) Transmission electron microscopic observation of mitochondria in control and shSEPT9 AGS cells in hypoxia (mitochondria: red arrows). Scale bars, 500 nm.

Metabolic reprogramming was a hallmark of tumors. The aberrant expression of metabolic enzymes was the most straightforward contributor to metabolic alterations.^[^ ^15^, ^16^, ^17^ ^]^ To identify whether the acetyltransferase NAT10 directly mediated the mRNA ac4C modification of HK1, HK2, HKDC1, and PDK1, acRIP‐seq analysis was performed. The sequential analysis of ac4C peaks showed that typical CXXCXXCXX motifs were highly enriched within ac4C sites (**Figure** **3**A). The ac4C peaks were predominantly occurring within coding sequences (CDSs) and 3' untranslated regions (3'UTR) (Figure 3B,C). Additionally, examination of peak distributions across transcripts revealed that the majority of mRNAs possess one to three ac4C peaks (Figure 3D). However, further data analysis showed no significant ac4C peak fold changes in mRNA between NAT10 silencing and control cells for the above enzymes, indicating that NAT10‐mediated mRNA ac4C modification may regulate the expression of these enzymes indirectly.

As reported, the acetyltransferase NAT10 conferred enhanced mRNA stability, and ac4C peaks within wobble sites stimulated translation efficiency.^[^ ^26^ ^]^ We therefore investigated potential targets using a combination of acRIP‐seq and Ribo‐seq. We identified seven candidate genes that showed concomitant decreased mRNA acetylation and reduced translation efficiency in shNAT10 cells (Figure 3E). It has been reported that SEPT9 upregulates HIF‐1*α* by blocking RACK1 activity in prostate cancer.^[^ ^42^ ^]^ Increased expression of HIF‐1*α* can activate the transcription of HK and PDK, which further initiates glucose metabolism reprogramming.^[^ ^43^ ^]^ Therefore, NAT10 might upregulate SEPT9 expression through mRNA ac4C modification and form a NAT10/SEPT9/HIF‐1*α* positive feedback loop that constantly over‐activates transcription of glycolytic enzymes downstream of the HIF‐1 pathway, consequently promoting glycolysis addiction of GC cells in a hypoxic microenvironment.

To substantiate this hypothesis, we thus chose SEPT9 as a candidate ac4C target in the regulation of metabolic shift for further studies. Significantly increased expression of SEPT9 in gastrointestinal carcinomas except colorectal cancer (CRC) was found by the TCGA and GEO datasets (Figure S4A, Supporting Information). Similarly, SEPT9 was frequently upregulated in GC tissues compared to normal gastric mucosa (Figure S4B, Supporting Information). Furthermore, SEPT9 mRNA was inversely associated with the survival of GC patients from KMplot (http://kmplot.com) datasets (Figure S4C, Supporting Information). Our data confirmed that SEPT9 was involved in regulating the reprogramming of glucose metabolism. SEPT9 silencing significantly attenuated glycolysis in GC cells (Figure 3F,G). Transmission electron microscopy showed that the mitochondria of GC cells were impaired in hypoxia, with the control group showing obvious mitochondrial swelling, while the shSEPT9 group showed less mitochondrial damage (Figure 3H). Consistent with these results, qPCR confirmed that SEPT9 knockdown was followed by a downregulation of HK1, HK2, HKDC1, and PDK1 (Figure S4D, Supporting Information). Similarly to NAT10 silencing, the proliferation and migration of shSEPT9 cells were attenuated (Figure S4E,F, Supporting Information). Altogether, these findings supported the possibility that SEPT9 was the downstream effector molecule in the ac4C‐regulated glycolysis. Furthermore, a NAT10/SEPT9/ HIF‐1*α* positive feedback loop may be formed to trigger overactivation of HIF‐1 signaling and glycolysis addiction in hypoxia.

### 2.4 ac4C Regulates mRNA Stability and Translation Efficiency of SEPT9

**Figure 4:**
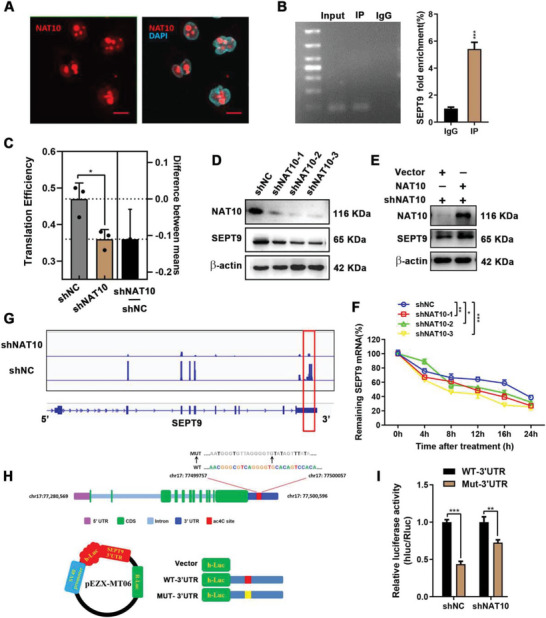
ac4C regulates the mRNA stability and translation of SEPT9 in GC cells. A) Immunofluorescence showed that NAT10 was predominantly distributed in the nucleus (NAT10, red; DAPI, blue). Scale bars, 10 µm. B) NAT10 RIP‐qPCR analysis of SEPT9 mRNA in AGS cells. C) Translation efficiency analysis of SEPT9 mRNA using Ribo‐seq. D,E) NAT10 promoted the protein expression of SEPT9. F) the mRNA levels of SEPT9 were detected in shNAT10 AGS cells after treatment with Act‐D for the indicated times. G) Integrative Genomics Viewer (IGV) tracks revealed the results of ac4C‐seq read distribution in SEPT9 mRNA of NAT10 knockdown and control AGS cells. Square marked decreased ac4C peaks in shNAT10 AGS cells. H) Schematic representation of positions of ac4C motifs within SEPT9 mRNA (upper panel). The ac4C sites within 3″UTR of SEPT9 mRNA were mutated to remove as many ac4C sites as possible. Schematic representation of mutated 3″UTR of pEZX‐MT06 vector to investigate the roles of ac4C in 3″UTR in SEPT9 mRNA stability (lower panel). I) shNC or shNAT10 AGS cells were transfected with WT or Mut 3″UTR reporters for 24 h.

To determine the relationship between ac4C modification and SEPT9 expression, we first performed IF to localize the cellular distribution of NAT10. We discovered NAT10 was predominantly located in the nucleus, implying that NAT10‐mediated mRNA ac4C modifications occur there (**Figure** **4**A). NAT10‐RIP followed by qPCR confirmed ac4c enrichment on SEPT9 mRNA (Figure 4B). As previously described, codon bias within CDS‐localized ac4C peaks promoted gene translation.^[^ ^26^ ^]^ Ribo‐seq and western blot confirmed that knockdown of NAT10 expression resulted in decreased translation efficiency and protein expression of SEPT9 (Figure 4C,D). In contrast, SEPT9 protein expression was restored when overexpressing NAT10 (Figure 4E).

Increasing mRNA stability was another major function of NAT10‐mediated ac4C modification.^[^ ^26^ ^]^ The half‐life of SEPT9 mRNA was ≈16 h, and reduced ac4C enrichment was accompanied by increased decay of SEPT9 mRNA (Figure 4F). To identify the key ac4C sites that regulate mRNA stability, we further analyzed the acetylation peaks of SEPT9 mRNA. acRIP‐seq data showed that the ac4C peaks were distributed in the CDS and 3″UTR region of SEPT9 mRNA (Figure 4G). However, the enrichment of the ac4C peak in the 3″UTR was significantly reduced in shNAT10 cells (fold change >2, FDR value <0.05), suggesting that this ac4C site may be more dynamic in regulating SEPT9 mRNA stability. We then constructed 3″UTR reporters containing wild type or mutant SEPT9 3″UTR after the firefly luciferase reporter gene (Figure 4H). Cytosine (C) was replaced with thymine (T) in ac4C peak of the mutant SEPT9 3′UTR. The dual‐luciferase assay showed attenuated fluorescence in the mut‐3″UTR groups, mirroring reduced mRNA stability due to the loss of acetylated C (Figure 4I). Overall, NAT10 promoted SEPT9 mRNA stability and translation efficiency via ac4C modification, and the ac4C peak within the 3″UTR region was responsible for mRNA stability.

### 2.5 A NAT10/SEPT9/HIF‐1*α* Positive Feedback Loop Exacerbates Glycolysis Addiction in GC Cells

**Figure 5:**
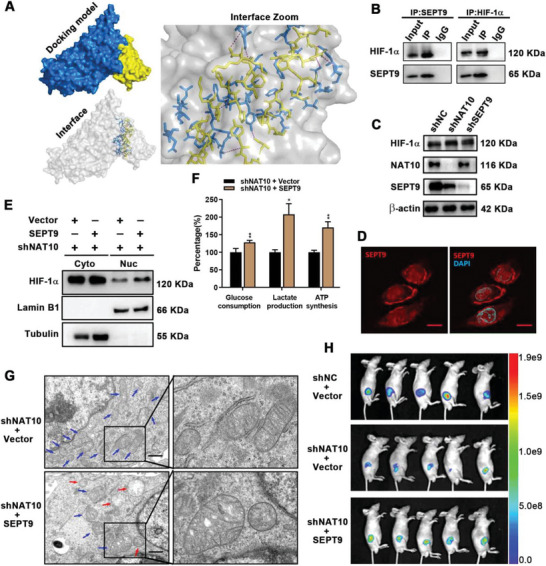
ac4C promotes glycolysis addiction via a NAT10/SEPT9/ HIF‐1*α* positive feedback loop. A) Surface diagram of the docking model and their interfacing residues between SEPT9 and HIF‐1*α* protein (SEPT9, blue; HIF‐1*α*, yellow; hydrogen bond interaction, dotted line). B) Immunoprecipitation analysis of the interaction between SEPT9 and HIF‐1*α* in AGS cells. C) Western blot analysis of the expression of HIF‐1*α* in shNAT10 or shSEPT9 AGS cells (with 100 µm CoCl_2_ treatment). D) AGS cells were grown and subjected to immunofluorescence staining (SEPT9, red; DAPI, blue) and confocal microscopy. Scale bars, 10 µm E) shNAT10+SEPT9 AGS cells were subjected to nuclear and cytoplasmic proteins extraction. F) Enhanced glucose consumption, lactate production, and ATP synthesis were observed in shNAT10+SEPT9 AGS cells (treated with 100 µm CoCl_2_). G) Transmission electron microscopic observation of mitochondria in control and shNAT10+SEPT9 AGS cells (treated with 100 µm CoCl_2_) in hypoxia (mitochondria, blue, and red arrows; Vacuolated mitochondria, red arrows). Scale bars, 1 µm. H) Whole‐body fluorescence images of xenograft GC nude mouse model.

SEPT9 is a member of the highly conserved GTP‐binding cytoskeleton protein family that is involved in various functions, including cell migration, cell cycle control, and oncogenesis. Its expression was positively associated with the growth kinetics and motility of tumor cells in breast,^[^ ^44^ ^]^ ovarian,^[^ ^45^ ^]^ and prostate cancers.^[^ ^42^ ^]^ Nevertheless, the mechanism of SEPT9 promoting the expression of hexokinase (HK1, HK2, HKDC1) and pyruvate dehydrogenase kinase (PDK1) remained obscure. Sharon Amir et al. reported that SEPT9 prevented the ubiquitination and degradation of HIF‐1*α* mediated by RACK1.^[^ ^42^ ^]^ To verify the relationship, we performed rigid protein–protein docking between SEPT9 and HIF‐1*α*. As shown in **Figure** **5**A and Table S5 (Supporting Information), SEPT9 and HIF‐1*α* formed hydrogen bonds through amino acid residue sites such as GLY 78‐PHE 295 and VAL 79‐THR 296, revealing that proteins SEPT9 and HIF‐1*α* formed a stable protein docking model. Furthermore, we found that SEPT9 interacted with HIF‐1*α* by Co‐IP (Figure 5B). Curiously, there was no significant decrease in total protein levels of HIF‐1*α* in NAT10 or SEPT9 knockdown GC cells (with 100 µm CoCl_2_ treatment) (Figure 5C). This suggested that other mechanisms may be present for SEPT9 to promote HIF‐1 pathway activation.

Intriguingly, IF analysis showed that SEPT9 accumulated abundantly in the GC cell nucleus (Figure 5D; Figure S5A, Supporting Information). Previous studies report that SEPT9 reinforced dynein tethering to the membrane cargo and/or microtubules, facilitating movement under high load.^[^ ^46^, ^47^ ^]^ SEPT9 may function as a transporter to influence the cellular localization of HIF‐1*α*. We thus extracted nucleus and cytoplasmic proteins separately to detect the cellular distribution of HIF‐1*α*. As predicted, knockdown of NAT10 or SEPT9 expression was accompanied by a decrease in intranuclear HIF‐1*α* (Figure S5B, Supporting Information). Moreover, nuclear HIF‐1*α* was reverted after overexpression of SEPT9 on the basis of NAT10 silencing (Figure 5E). Consistent with increased metabolic enzymes (HK1, HK2, HKDC1, and PDK1) expression, the glycolysis in shNAT10+SEPT9 GC cells was synchronously upregulated (Figure 5F; Figure S5C, Supporting Information). Compared with the control cells, shNAT10+SEPT9 GC cells in hypoxia had obvious mitochondrial dysfunction, with a swollen morphology, shortened or even absent mitochondrial cristae, and some mitochondria showed focal vacuolation or transformation into small vacuolated structures (Figure 5G).

Additionally, shNAT10+SEPT9 GC cells showed more dynamic proliferation in vivo (Figure 5H; Figure S5D,E, Supporting Information). Bolstered by these results, it revealed a novel mechanism that NAT10/SEPT9/ HIF‐1*α* developed a positive feedback loop driving glycolysis addiction to sustain rapid energy synthesis for multiple cellular activities in a hypoxic environment.

### 2.6 Combining ac4C Inhibition with Apatinib Optimizes Anti‐Tumor Efficacy

Our data indicated that mRNA ac4C modification promoted glycolysis addiction through the NAT10/SEPT9/ HIF‐1*α* positive feedback loop, which facilitated the energy synthesis and survival of GC cells in a hypoxic environment. Here, we assessed NAT10 inhibition as a potential therapeutic strategy for GC by Remodelin treatment. As described in previous researches,the specific inhibitor suppressed NAT10 function at concentrations that are non‐toxic in culture or in mice.^[^ ^27^, ^48^, ^49^ ^]^ Consistent with the shNAT10 cells, reduced RNA acetylation led to diminished glycolysis and less mitochondrial damage in Remodelin‐treated GC cells (Figure S6A–C, Supporting Information). It implied that Remodelin could effectively allow cancer cells to escape glycolysis dependency.

**Figure 6:**
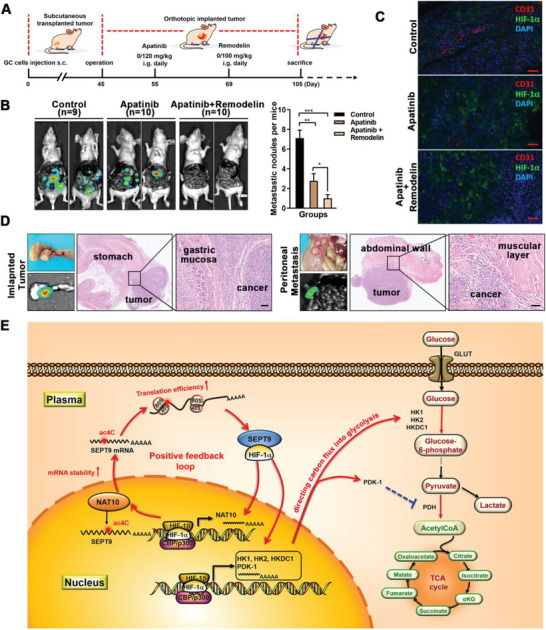
Combining ac4C inhibition with apatinib optimizes antitumor effects. A) Diagram depicting the construction processes and treatment schedule for the orthotropic xenograft GC mouse model. B) Fluorescence images of tumor metastasis in the orthotropic xenograft GC nude mouse models (left panel). The numbers of metastatic nodules (hepatic, intestinal, and peritoneal nodules) per group were quantitatively analyzed (right panel). C) Dual staining of CD31 and HIF‐1*α* to assay angiogenesis and hypoxic changes in the implanted tumors. (CD31, red; HIF‐1*α*, green; DAPI, blue). Scale bars, 50 µm. D) Representative images of implanted tumors and metastatic nodules (indicated by red circles or fluorescence) were shown and detected by hematoxylin‐eosin (H&E) staining. Scale bars, 50 µm. E) Schematic diagram depicting the mechanism of NAT10/SEPT9/HIF‐1*α* positive feedback loop regulating glycolysis addiction in GC cells.

Anti‐angiogenic therapy reduced tumor blood supply, leading to a hypoxic and acidotic tumor microenvironment. Due to the Warburg effect, cancer cells rewired glucose metabolism to promote survival, proliferation, and long‐term maintenance.^[^ ^50^, ^51^, ^52^ ^]^ Therefore, we hypothesized that attenuating hypoxia tolerance in GC cells by ac4C inhibition may enhance therapeutic efficacy when combined with anti‐angiogenic therapy. We then constructed orthotropic xenograft GC mouse models according to our previous study for further investigation.^[^ ^53^, ^54^ ^]^ On day 55, apatinib (120 mg kg^−1^, oral gavage, daily) was used to induce hypoxia in the tumor, followed by Remodelin (100 mg kg^−1^, oral gavage, daily) treatment two weeks later (**Figure** **6**A). Mice were dissected to observe tumor growth and metastasis in the abdominal cavity, when they reached the end point. As expected, tumors in the control group developed significant adhesions to the adjacent organs and multiple tumor metastases (liver, intestine, and peritoneum) (Figure 6B,D; Figure S6D, Supporting Information). Apatinib markedly inhibited tumor metastasis, and this anti‐tumor efficacy was further potentiated by the combination of Remodelin (Figure 6B). The dual staining of CD31 and HIF‐1*α* showed that tissue hypoxia was observed in the implanted tumors. It was exacerbated when applying apatinib for anti‐angiogenesis (Figure 6C). Thus, the combination of anti‐angiogenesis and inhibition of glycolysis addiction might reduce the hypoxia tolerance of tumor cells, which prevented them from responding to microenvironmental stress. Altogether, these results indicated that combining ac4C inhibition with apatinib is a promising therapeutic strategy to inhibit GC progression.

## 3 Discussion

Alterations to the epigenome occur in almost all human cancers.^[^ ^55^ ^]^ Unique and reversible epigenetic states in tumor cells may promote metastasis, lead to drug resistance, or predict patient outcome. As a recent hot topic of epigenetic area, studies on the biological functions of mRNA ac4C modification have sprung up.^[^ ^27^, ^28^, ^29^, ^30^, ^31^, ^32^, ^33^, ^34^, ^35^ ^]^ Consistent with previous studies,^[^ ^35^ ^]^ we also observed that acetyltransferase NAT10 expression was upregulated in GC and positively correlated with a poor prognosis in GC patients. More importantly, we revealed that ac4C modification promotes resistance to anti‐angiogenic therapy through enhancing the hypoxia tolerance of GC cells. We identified that HIF‐1*α* interacted with the HRE on NAT10 promoter, resulting in more active NAT10 transcription in hypoxia. Since hypoxia is a common feature of solid tumors, the correlation between HIF‐1*α* and NAT10 may provide a clear explanation for the substantial enrichment of NAT10 in multiple tumor tissues.

Strikingly, the combined analysis of acRIP‐seq, Ribo‐seq, and RNA‐seq confirmed that NAT10 in turn promoted HIF‐1*α* translocation into the nucleus via SEPT9 mRNA ac4C modification, followed by enhanced transcription of metabolic enzymes (HK1, HK2, HKDC1, and PDK1). Consequently, a NAT10/SEPT9/ HIF‐1*α* positive feedback loop was constituted, triggering continuous overactivation of HIF‐1 pathway and subsequent metabolic rewiring toward a glycolytic phenotype (Figure 6E). Here, we emphasized the critical regulatory role of mRNA ac4C modification in driving HIF‐1 mediated glycolysis addiction, identifying NAT10 as a potential therapeutic target for cancer therapy.

Interestingly, SEPT9, a member of the highly conserved septin family, exhibited distinct expression features in several common gastrointestinal tumors (Figure S3A, Supporting Information). It has been shown that hypermethylation of SEPT9 promoter regions was detected in tumor tissue and plasma of CRC patients, which was proposed to be a sensitive and specific biomarker for non‐invasive CRC screening.^[^ ^56^, ^57^, ^58^ ^]^ Thus, SEPT9 was once regarded as a tumor suppressor. However, pan‐cancer analysis showed that SEPT9 expression was increased in esophageal, gastric, and hepatic cancers. The combined analysis of acRIP‐seq and Ribo‐seq confirmed that mRNA ac4C modification contributed to SEPT9 overexpression in GC. As described in previous studies, SEPT9 also functions as an oncogene.^[^ ^42^, ^44^, ^59^, ^60^ ^]^ For example, Fernando Calvo et al. discovered that SEPT9‐mediated F‐actin bundling promoted amoeboid melanoma cell migration and invasion by stabilizing highly contractile actomyosin structures.^[^ ^59^ ^]^ Additionally, SEPT9 protected HIF‐1*α* from degradation by preventing the interaction between HIF‐1*α* and RACK1, and induced angiogenesis in prostate cancer.^[^ ^42^ ^]^ In the present study, we did detect the interaction of SEPT9 with HIF‐1*α* by molecular docking analysis and Co‐IP, but we observed a reduced accumulation of HIF‐1*α* in the nucleus rather than a decrease in total HIF‐1*α* protein levels when interfering with NAT10 or SEPT9 expression. This suggested that SEPT9 served as a vehicle to facilitate the translocation of HIF‐1*α* into the nucleus in GC cells. Different tumor backgrounds may be responsible for the functional differences of SEPT9. Here, we demonstrated that the interaction of SEPT9 with HIF‐1*α* is an essential process in the glucose metabolism shift regulated by ac4C.

As a hallmark of cancer cells, glucose metabolism reprogramming facilitates the adaptation of cancer cells to the hypoxic microenvironment and competition for limited nutritional resources.^[^ ^15^, ^16^, ^17^, ^51^ ^]^ Additionally, increasing evidence has revealed that this aberrant glucose metabolic behavior contributes to immune escape and microenvironmental remodeling.^[^ ^61^, ^62^, ^63^ ^]^ Hence, therapeutic targeting of glycolysis has been an attractive proposition since the discovery of metabolic reprogramming. Although glycolysis dependence induced by glucose metabolic reprogramming is substantially elevated in various tumors, therapeutic targeting of glycolysis in cancer patients has not yet been successful. Anti‐glycolytic agents (e.g., 2‐DG and Lonidamine) as monotherapy have failed to show anti‐tumor efficacy in clinical trials.^[^ ^18^ ^]^ It indicated that improvement based on clinical therapy protocols may be an appropriate strategy. Therefore, we focused on apatinib, which has been shown to yield significant therapeutic benefit and is the first anti‐angiogenic agent approved by the cFDA for the treatment of refractory GC.^[^ ^5^, ^6^ ^]^ As an inhibitor of angiogenesis, apatinib exacerbates hypoxia in tumor tissue and results in an overdependence on glycolysis of tumor cells to cope with increased metabolic and survival stress.^[^ ^9^, ^10^ ^]^ It is consistent with our findings that a NAT10/SEPT9/HIF‐1*α* positive feedback loop regulates glycolysis addiction. Additionally, we found that VEGFA, an essential HIF‐1 target gene responsible for angiogenesis, was downregulated in shNAT10 GC cells (Figure 2D). These results suggested that ac4C inhibition may hinder the reprogramming of glucose metabolism in GC while inhibiting angiogenesis, which enhances the antitumor efficacy of apatinib. Meanwhile, inhibitors targeting mRNA modifying enzymes have been successfully applied in acute myeloid leukemia (AML) treatment, which was considered as a promising new avenue of anti‐cancer therapy.^[^ ^64^ ^]^ Therefore, Remodelin, a specific ac4C inhibitor, may be a suitable candidate for overcoming resistance to anti‐angiogenic therapy. As expected, our data demonstrated the anti‐tumor efficacy of this combination therapy. It is a complementary treatment strategy. For one thing, ac4C inhibition disrupts the metabolic reprogramming induced by hypoxia. For another, anti‐angiogenesis treatment exacerbates intra‐tumor hypoxia, which enhances the targeting of ac4C inhibitors.

In conclusion, our research uncovered a correlation between mRAN ac4C modification and resistance to anti‐angiogenic therapy, and a novel mechanism that was responsible for the overexpression of NAT10 in GC. Furthermore, we emphasized the crucial role of the NAT10/SEPT9/HIF‐1*α* positive feedback loop in regulation of glycolysis addiction and provided compelling in vitro and in vivo evidence demonstrating that targeting mRNA ac4C modification could be a promising therapeutic strategy for GC treatment.

## 4 Experimental Section

### Cell Lines and Human Tissues

Human GC cell lines (AGS and MGC 80–3) were obtained from the Type Culture Collection of the Chinese Academy of Sciences (Shanghai, China). Cell lines were routinely cultured and maintained in RPMI 1640 (Gibco) supplemented with 10% fetal bovine serum (Gibco) and 1% penicillin‐streptomycin (Gibco) within a humidified incubator containing 5% CO_2_ at 37 °C. Cobalt chloride (CoCl_2_; Sigma‐Aldrich, St Louis, MO, USA) was used to simulate hypoxic conditions as previously reported.^[^ ^65^, ^66^ ^]^ Briefly, cells were treated with 100 µm CoCl_2_ and incubated for 8–24 h.

The GC tissues and normal gastric mucosa tissues (*n* = 78) were acquired from consenting patients undergoing surgery in the Nanfang Hospital of Southern Medical University. This study was reviewed and approved by the Ethics Committee of Nanfang Hospital (Guangzhou, China) (NCT01609309). The experiments with human subjects were carried out with the full, informed consent of the subjects.

### Construction of Stable Knockdown and Overexpressed Cells

The lentiviral vectors plasmids (shNAT10, shSEPT9, NAT10‐OE, and SEPT9‐OE) were purchased from Genecopoeia. Negative controls included the shNC plasmid or an empty vector. AGS and MGC 80–3 were chosen to establish stable gene knockdown or overexpression models with the Lenti‐Pac HIV Expression Packaging Kit (Cat# LT002, Genecopoeia). The plasmid information is listed in Table S1 (Supporting Information).

### RNA Extraction and Real‐Time PCR Analysis

Total RNA from target cells was isolated with the Trizol reagent (Invitrogen), followed by reverse transcription for purified cDNA templates. The qRT‐PCR was performed with a TB Green Premix Ex Taq II Kit (Cat# RR820A, Takara) according to the manufacturer's recommendation, and the primers of targeted genes are listed in Table S2 (Supporting Information). Transcripts of *β*‐actin were used for normalization.

### Western Blotting Analysis

Western blot analysis was performed as described in the previous study.^[^ ^53^, ^54^ ^]^ Briefly, proteins from target cells were separated by SDS‐PAGE and then transferred onto polyvinylidene fluoride (PVDF) membranes as described. The corresponding quantitative analysis of western blot results is shown in Figure S7 (Supporting Information). The primary antibodies used are shown in Table S3 (Supporting Information).

### ac4C Detection by Dot Blot

Dot blots were performed using rabbit monoclonal anti‐ac4C antibodies as described previously.^[^ ^26^ ^]^ Briefly, 5–10 µg RNA were denatured at 75 °C for 5 min and immediately chilled on ice for 1 min. The products were loaded onto the nylon membranes (0.45 µm, Biodyne‐PALL). The membranes were then cross‐linked twice with a Stratalinker 2400 UV Cross‐linker in the autocrosslink mode (1200 microjoules [x100], 25–50s), blocked for 1 h at room temperature with 5% nonfat milk in 0.1% PBST, and incubated overnight at 4 °C with the anti‐ac4C antibody (Cat#ab252215, Abcam, 1:1000 dilution). After washing the membranes three times for 5 min, they were incubated for 1 h at room temperature with anti‐rabbit IgG‐HRP (1:10000 dilution), washed three times, and exposed with Hyperfilm ECL for the appropriate exposure period.

### Metabolic Assay

The glucose and lactate concentrations in cultured media were measured using commercial kits (Cat#MAK263 and MAK064, Sigma‐Aldrich) following the manufacturer's instructions. The enhanced ATP assay kit (Cat #S0027, Beyotime Biotechnology) was used to quantify cellular ATP according to the manufacturer's instructions. All samples were detected in triplicate.

### mRNA Stability Assay

Target cells were incubated in complete RPMI 1640 medium containing 5 µg ml^−1^ actinomycin D (Cat #A9415, Sigma) for 0, 4, 8,12,16, and 24 hrs. Cells were collected at the indicated times, and total RNA was isolated as described in the section “RNA extraction” for real‐time PCR.

### Mito Tracker Staining

To observe the change in mitochondrial membrane potential, target cells were stained with Mito Tracker Red CMXRos (Cat #C1049B, Beyotime Biotechnology) at a final concentration of 50 nm for 30 min in the dark. The cells were then washed three times with prewarmed PBS and replaced with fresh medium. Cellular mitochondrial fluorescence intensity was observed by confocal laser microscopy.

### Transmission Electron Microscopy Analysis

The GC cell samples were fixed with 2.5% glutaraldehyde for 3 h at 4 °C. Following three washes, the samples were post‐fixed with 1% OsO_4_ for 1 h before being dehydrated in sequential ethanol solutions (30, 50, 70, 90, and 100%). Then, the samples were embedded in Eponate mixture (Electron Microscopy Sciences, Hatfield, PA) for polymerization. The ultrathin sections were then photographed with a transmission electron microscope.

### Luciferase Reporter Gene Assay

The promoter activity of NAT10 in GC cells was measured by luciferase assay. Briefly, GC cells were treated with 0–250 µm CoCl_2_ and transfected with pEZX‐PL01‐NAT10‐WT‐Luc or pEZX‐ PL01‐NAT10‐Mut‐Luc containing the −2000/+1 sequence of the NAT10 promoter for 24 h.

To evaluate the effect of ac4C peak within the 3″UTR on SEPT9 mRNA stability, the mutant or wild type 3″UTR of SEPT9 was inserted behind the h‐luc coding region. Both the pEZX‐MT06 ‐SEPT9‐3″UTR ‐WT and pEZX‐MT06 ‐SEPT9‐3″UTR ‐Mut were transfected into shNC or shNAT10 AGS cells for 24 h, the firefly luciferase and renilla luciferase were assayed by Duo‐Luciferase HS Assay Kit (Cat# LF004, GeneCopoeia) according to the manufacturer's instructions. The plasmid information is listed in Table S1 (Supporting Information).

### NAT10‐RIP (RNA Immunoprecipitation)

The RIP assay was carried out to confirm the interaction between NAT10 and SEPT9 mRNA using RIP Kit (Cat# Bes5101, BersinBio). Briefly, cells were lysed by the polysome lysis buffer containing protease inhibitor and RNase inhibitor. DNase was added to the cell lysate and incubated at 37 °C for 10 min to degrade the DNA. One microgram NAT10 or IgG antibodies were added to the samples and incubated at 4 °C for 16 h in a vertical mixer. After that, the samples were incubated for 1 h with protein A/G beads. Following the manufacturer's instructions, the samples were washed with polysome washing buffer and treated the beads containing the immunoprecipitated RNA‐protein complex with proteinase K to remove proteins. The interested RNAs were then extracted using the phenol‐chloroform method and detected using RT‐qPCR with normalization to their input group.

### Acetylated RNA Immunoprecipitation Sequencing (acRIP‐seq)

acRIP‐seq and data analysis were done by Guangzhou Epibiotek Co., Ltd. The shNC and shNAT10 GC cells were subjected to acRIP‐seq, and three independent biological replicates of each group were performed. Briefly, total RNA was extracted and purified from shNAT10 or shNC GC cells using TRIzol reagent (Invitrogen). Hundred microgram total RNA was fragmented into 100–200 nt RNA fragments using 10X RNA Fragmentation Buffer (100 mm Tris‐HCl, 100 mm ZnCl_2_ in nuclease‐free H_2_O). The reaction was stopped by adding 10X EDTA (0.5 m EDTA). To obtain immunoprecipitated RNA fragments, fragmented RNA was incubated for 3 h at 4 °C with anti‐ac4C monoclonal antibody and then for 2 h at 4 °C with protein A/G magnetic beads (Cat# 8880210002D/10004D, Invitrogen) according to the Epi^TM^ ac4C immunoprecipitation kit (Epibiotek, R1815). The library was prepared using the smart‐seq method. Both the input samples without IP and the ac4C IP samples were subjected to 150‐bp, paired‐end sequencing on an Illumina NovaSeq 6000 sequencer.

### Ribosome Profiling Sequencing (Ribo‐seq)

Ribo‐seq and data analysis were done by Guangzhou Epibiotek Co., Ltd. The shNC and shNAT10 GC cells were subjected to Ribo‐seq, and three independent biological replicates of each group were performed. Briefly, shNAT10 or shNC GC cells were treated with 100ug ml^−1^ cycloheximide. According to the Epi™ Ribosome Profiling Kit (Cat# R1814, Epibiotek), the target cells were lysed and incubated with Ribo‐Seq Nuclease Mix. The reaction was stopped by adding SUPERase to the RNase Inhibitor (Cat#AM2696, Life Technologies). Then, RNA was collected with the RNA Clean&Concentrator‐5 kit (Cat#R1016, ZYMO) and subjected to rRNA depletion using the EpiTM RiboRNA Depletion Kit (Cat# R1805, Epibiotek) according to the manufacturer's instructions. The recovered RNA was prepared for library construction and subsequent quality control using the Bioptic Qsep100 Analyzer (Bioptic lnc).

### RNA Sequencing (RNA‐seq)

RNA ‐seq and data analysis were performed by Guangzhou Epibiotek Co., Ltd. The shNC and shNAT10 GC cells were subjected to RNA‐seq, and three independent biological replicates of each group were performed. VAHTS Stranded mRNA‐seq Library Prep Kit for Illumina V2 (Cat#NR612‐02, Vazyme Biotech) was used for library preparation according to the instructions, followed by the computational analysis they provided.

### Co‐Immunoprecipitation (Co‐IP)

Target cells were treated with 100 µm CoCl_2_ for 8 h and then total cellular proteins were extracted with cell lysis buffer containing protease inhibitor and phosphatase inhibitor. To verify the interaction between SEPT9 and HIF‐1*α*, lysate was incubated with anti‐SEPT9 or IgG at 4 °C overnight, followed by incubation with protein A/G magnetic beads for 4–6 h at 4 °C. The immunoprecipitated protein complexes were isolated from the beads after several washes, and then the interaction between SEPT9 and HIF‐1*α* was identified by western blot.

### Molecular Docking Analysis

Rigid protein–protein docking was performed between SEPT9 and HIF‐1*α* to investigate the relationships by using GRAMM‐X (http://gramm.compbio.ku.edu/). The protein structural domains of SEPT9 and HIF‐1*α* were obtained from the Protein Data Bank PDB database (http://www.rcsb.org/). Pymol (Version 2.4) and PDBePISA (https://www.ebi.ac.uk/pdbe/pisa/) were used to investigate protein‐protein interactions and further visual analysis.

### Tumor Growth Assay (Subcutaneous Tumor Model)

Athymic BALB/c nude mice (4 to 5 weeks old) were purchased from the Central Laboratory of Animal Science at Southern Medical University (Guangzhou, China) and raised in specific pathogen‐free conditions. The animal experiments were approved by the Institutional Animal Care and Use Committee (IACUC) of Nanfang Hospital (Guangzhou, China) (NFYY‐2019‐1133). MGC 80–3 cells were suspended in 200 µl PBS at a final concentration of 1 × 10^6^ cells and implanted subcutaneously into mice to investigate tumor growth. Tumor volumes were measured every three days and calculated as follows: tumor volume (mm^3^)  =  (Length × Width^2^)/2.

### Orthotropic Xenograft GC Nude Mice Model (Metastasis Tumor Model)

As described in the tumor growth assay, MGC 80–3 cells with EGFP were subcutaneously injected into the mice. An additional 30 nude mice were used to construct the orthotropic xenograft mouse model according to the previous study. Ten days after operation, the mice were randomly assigned into the apatinib, apatinib + Remodelin, or control groups (*n* = 10/group) and then treated daily with oral gavage of apatinib (120 mg/kg/day) (Cat#S5248, Selleck) or the same amount of DMSO. Remodelin (100 mg/kg/day) (Cat#S7641, Selleck) was administered by oral gavage every day in apatinib + Remodelin group, while the apatinib or control group was treated with the same amount of DMSO. The health status of mice (such as body weight, physical activity, and breath) was monitored. The mice were euthanized using cervical dislocation when reaching humane endpoints. The tumorigenesis in mice was observed by using the multi‐functional in vivo imaging system (Ami HT / Ami HTX Next Generation Preclinical Optical Imaging Systems, Spectral Instruments, USA). The primary tumors and metastatic nodules were resected for hematoxylin and eosin (H&E) staining.

### Immunofluorescence (IF) and Immunohistochemistry (IHC) Analyses

IF and IHC analyses were performed to assay the expression of target proteins according to theprevious study.^[^ ^53^, ^54^ ^]^ The antibodies used in this study are shown in Table S3 (Supporting Information).

### Cell Proliferation Assay

To assess the proliferation of cells in hypoxia, target cells were seeded in 96‐well plates at 1 × 10^4^ cells/well in complete medium containing 100 µm CoCl_2_. The CCK‐8 cell viability assay was performed according to the manufacturer's protocol for the indicated time.

### Wound Healing Assay

Five parallel lines were drawn on the underside of each well with a marker pen before seeding the indicated GC cells. Approximately 5 × 10^5^ cells per well were seeded into 6‐well plates. After cells had become adherent, five parallel scratches or “wounds” were made perpendicular to the marked lines using a 200 µl pipette tip. The migration of cells into the “wounds” was observed using an inverted microscope, and the images of areas flanking the intersections of the “wound” and the marked lines were taken at regular intervals over the course of 48 h.

### Statistical Analysis

All data were presented as mean ± SEM unless otherwise specified. The differences between two groups were assessed using a two‐tailed unpaired Student's t‐test. One‐way ANOVA was used to compare the means of three or more experimental groups. Statistical analyses were performed using GraphPad Prism 9.0. Statistical significance was defined as a *p*‐value of < 0.05. *p < 0.05, **p < 0.01, ***p < 0.001. NS, no significant.

## Conflict of Interest

The authors declare no conflict of interest.

## Supporting information

Supporting Information

## Data Availability

The data that support the findings of this study are available from the corresponding author upon reasonable request.
