# Supplementary material for: N4‐Acetylcytidine Drives Glycolysis Addiction in Gastric Cancer via NAT10/SEPT9/HIF‐1α Positive Feedback Loop
Source: Adv Sci (Weinh). 2023 Jun 16;10(23):2300898. doi: 10.1002/advs.202300898 (PMC10427357; doi:10.1002/advs.202300898)
Supplement: Supplementary file 1 — Supporting Information [file ADVS-10-2300898-s001.pdf]

ADVANCED SCIENCE
Open Access

Supporting Information

for *Adv. Sci.*, DOI 10.1002/advs.202300898

N4-Acetylcytidine Drives Glycolysis Addiction in Gastric Cancer via NAT10/SEPT9/HIF-1α Positive Feedback Loop

Qingbin Yang, Xuetao Lei, Jiayong He, Yanmei Peng, Yihao Zhang, Ruoyu Ling, Chaorui Wu, Guofan Zhang, Boyang Zheng, Xinhua Chen, Boya Zou, Ziyi Fu, Liying Zhao, Hao Liu, Yanfeng Hu, Jiang Yu, Fengping Li, Gengtai Ye* and Guoxin Li**

Supporting Information

Title

N4-acetylcytidine drives glycolysis addiction in gastric cancer via NAT10/SEPT9/HIF-1α positive feedback loop

Qingbin Yang#, Xuetao Lei#, Jiayong He#, Yanmei Peng#, Yihao Zhang, Ruoyu Ling, Chaorui Wu, Guofan Zhang, Boyang Zheng, Xinhua Chen, Boya Zou, Ziyi Fu, Fengping Li*, Gengtai Ye*, and Guoxin Li*

Supplementary data

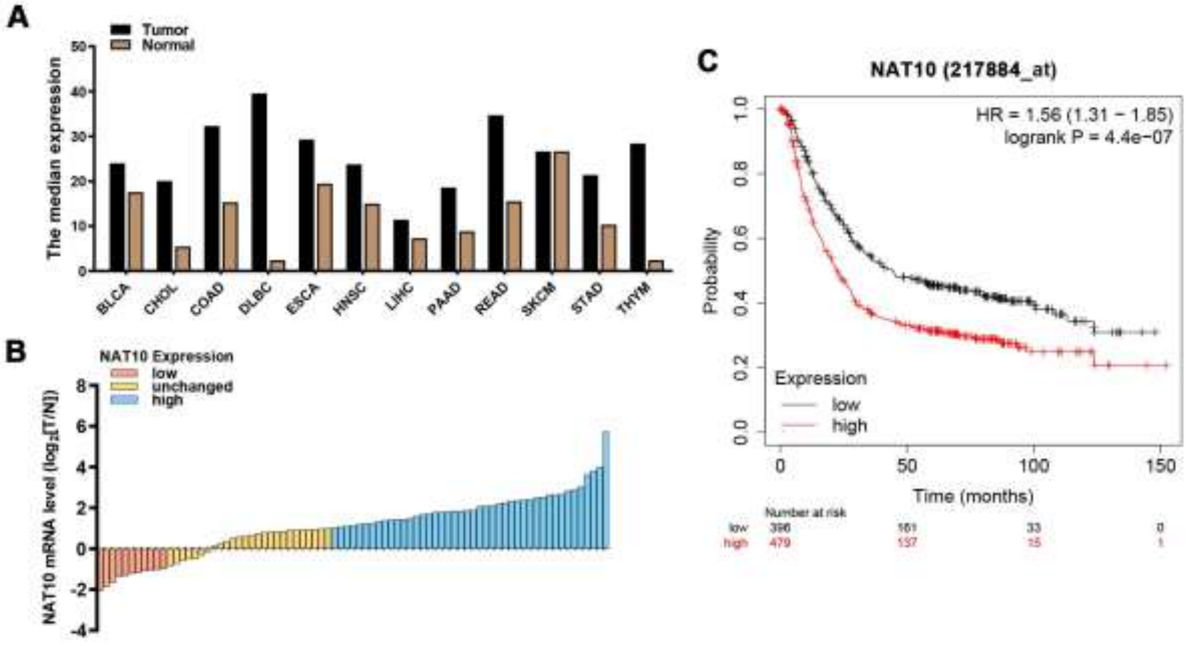

Figure S1. NAT10 is upregulated in GC.

(A) TCGA and GEO dataset analysis showed that SEPT9 expression was upregulated in a variety of common tumors, including gastric cancer (STAD; Tumor (n)=408, Normal (n)=211). (B) Relative NAT10 expression in 78 paired of GC and corresponding normal tissues measured by qPCR were shown using a waterfall plot. The fold change of relative NAT10 expression (log2[T/N]) >1 or <-1 was defined as significant. (C) The Kaplan-Meier survival analysis of overall survival in TCGA GC data based on NAT10 expression.

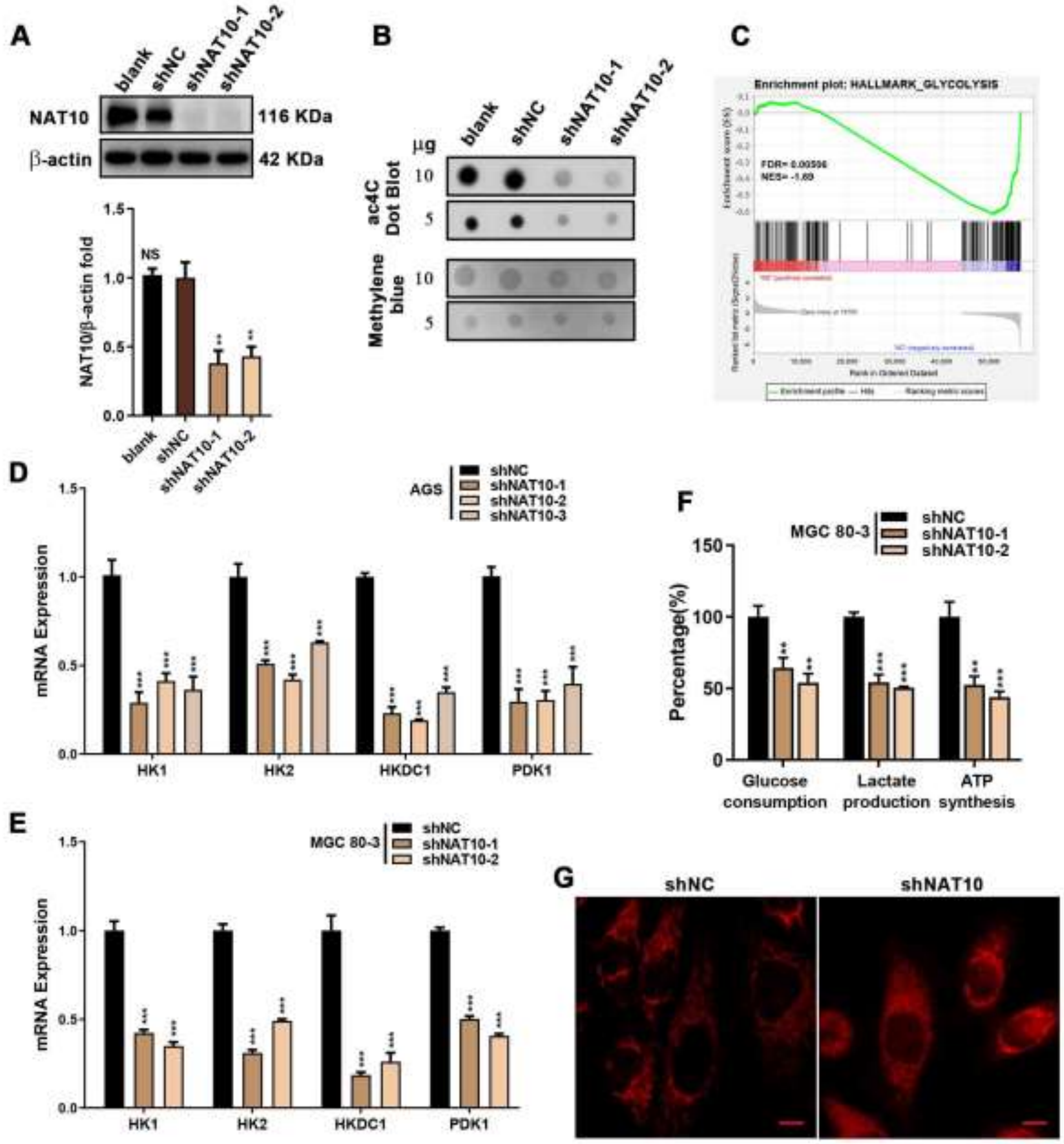

Figure S2. ac4C regulates glycolysis and ATP generation of GC cells.

(A) Expression of NAT10 in shRNAs transfected MGC 80-3 cells were analyzed by Western blot and qPCR. (B) The total RNA ac4C level was decreased in NAT10-knockdown MGC 80-3 cells. (C) GSEA revealed negative enrichment of genes in glycolysis sets of shNAT10 AGS cells. (D and E) qPCR analysis showed that the expression of HK1, HK2, HKDC1 and PDK1were down-regulated in AGS (D) or MGC 80-3 (E) GC cells with NAT10 knockdown. b-actin mRNA served as an internal control. (F and G) NAT10 knockdown MGC 80-3 cells exhibited lower levels of glycolysis (F) and less mitochondrial damage (G). The cells were treated with 100 μM $CoCl_2$. Scale bars, 10 μm.

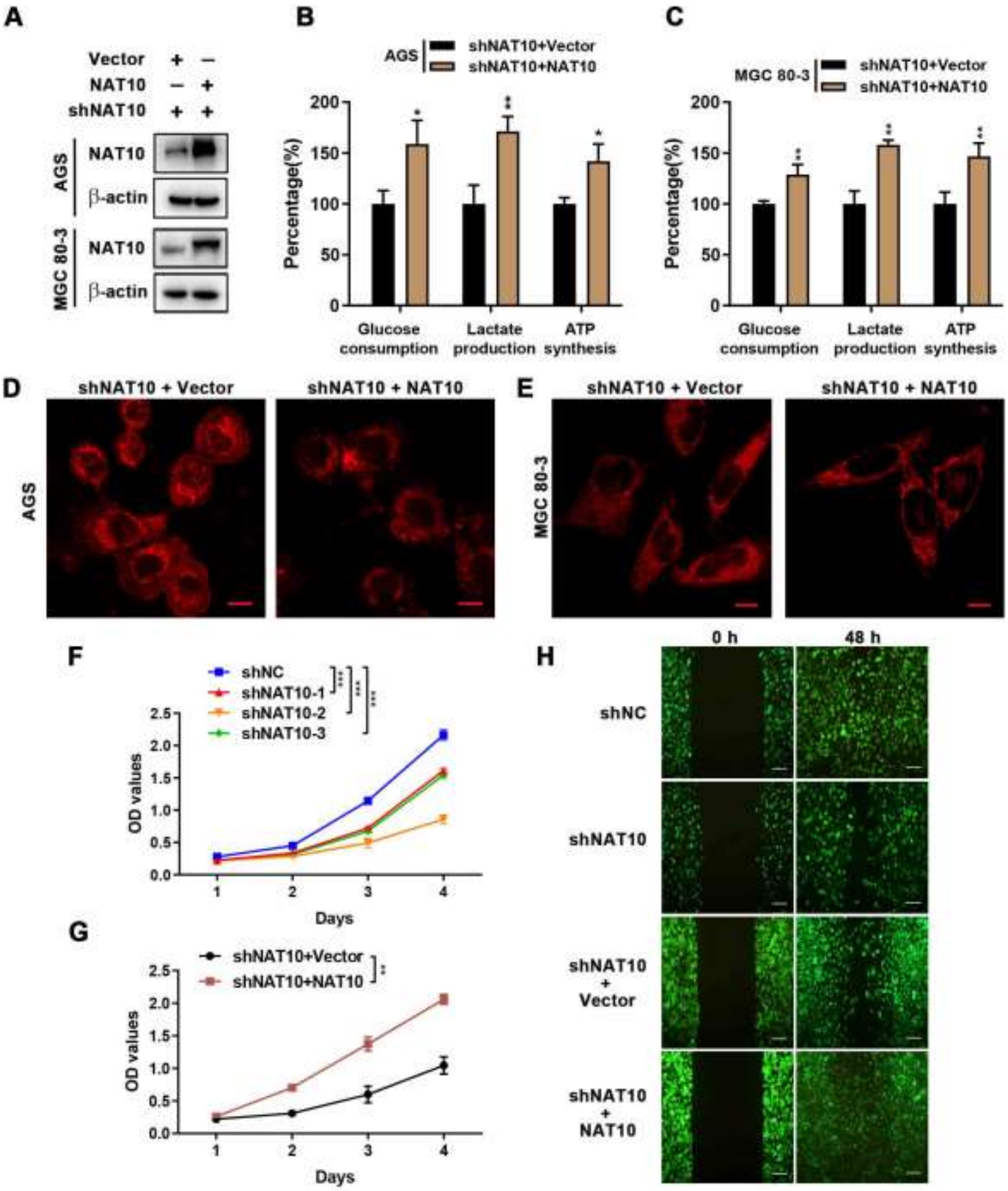

Figure S3. ac4C enhances proliferation and migration of GC cells by regulating glycolysis.

(A) Expression of NAT10 in shNAT10+NAT10 AGS and MGC 80-3 cells, as measured by Western blot. (B-H) The GC cells were treated with 100 µM $CoCl_2$. (B and C) Glycolysis of GC cells (B, AGS cells; C, MGC 80-3 cells) was restored by reversing NAT10 expression. (D and E) The mitochondria in shNAT10+NAT10 GC cells (D, AGS cells; E, MGC 80-3 cells) were depolarized along with decreased MMP. Scale bars, 10 µm. (F and G) The proliferation of NAT10 knockdown (F) or NAT10 overexpression (G) AGS cells were tested using CCK8

assay. (H) The migration changes of different AGS cells were tested using monolayer wound healing assay. Scale bars, 500 μm.

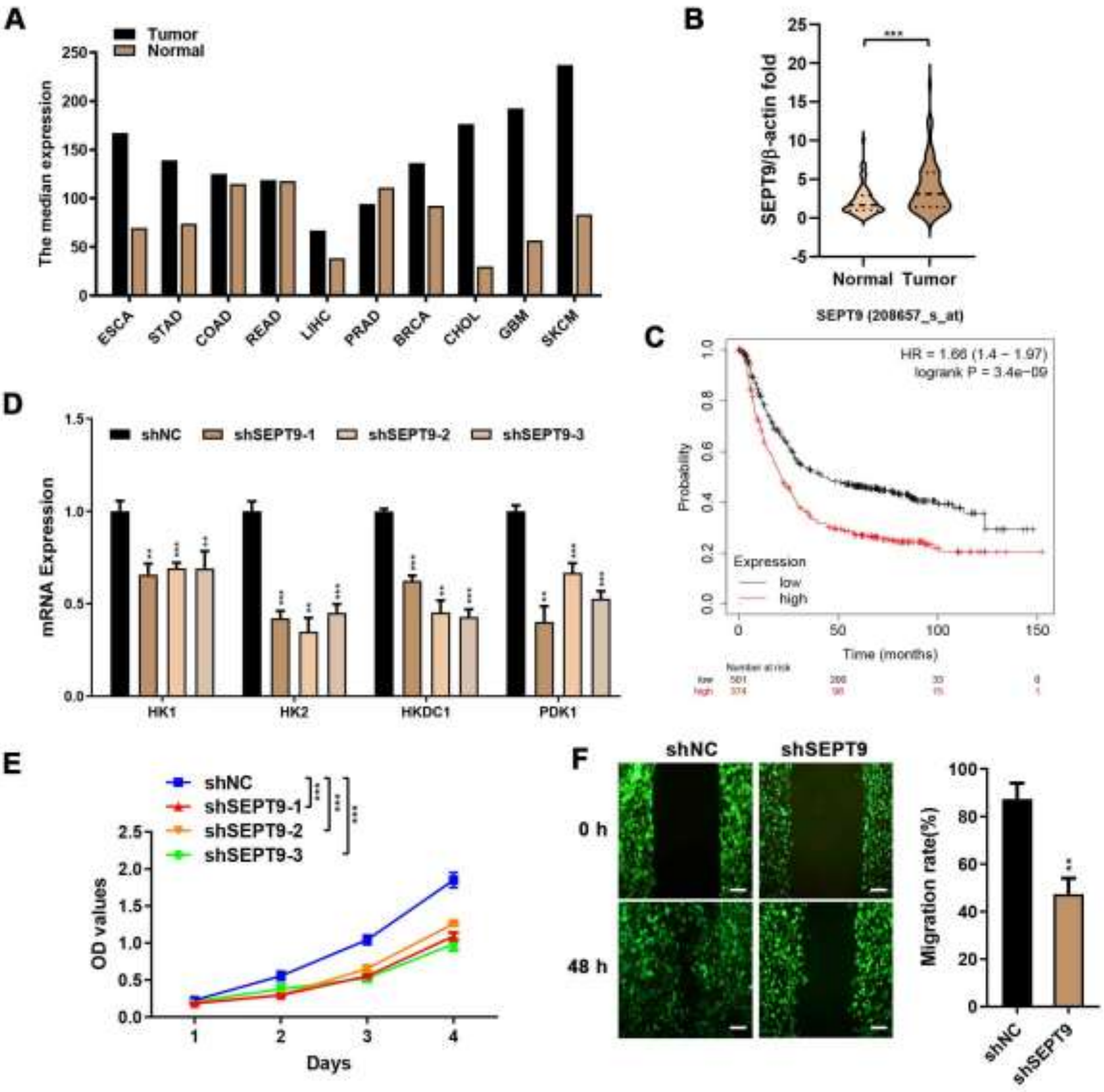

Figure S4. SEPT9 is upregulated in GC and associated with poor survival of GC patients.

(A) TCGA and GEO dataset analysis showed that SEPT9 expression was upregulated in multiple gastrointestinal tumors, including gastric cancer (Tumor (n)=408, Normal (n)=211). (B) Expression of SEPT9 was determined by qPCR in 78 paired of GC and corresponding normal tissues. (C) The Kaplan-Meier survival analysis of overall survival in TCGA GC data based on SEPT9 expression. (D) The transcription of HK1, HK2, HKDC1 and PDK1 in shSEPT9 AGS cells were detected by qPCR. (E and F) The proliferation and migration changes of SEPT9 knockdown AGS cells were tested using CCK8 assay (E) and monolayer

wound healing assay (F), respectively. The GC cells were treated with 100 μM $CoCl_2$.Scale bars, 500 μm.

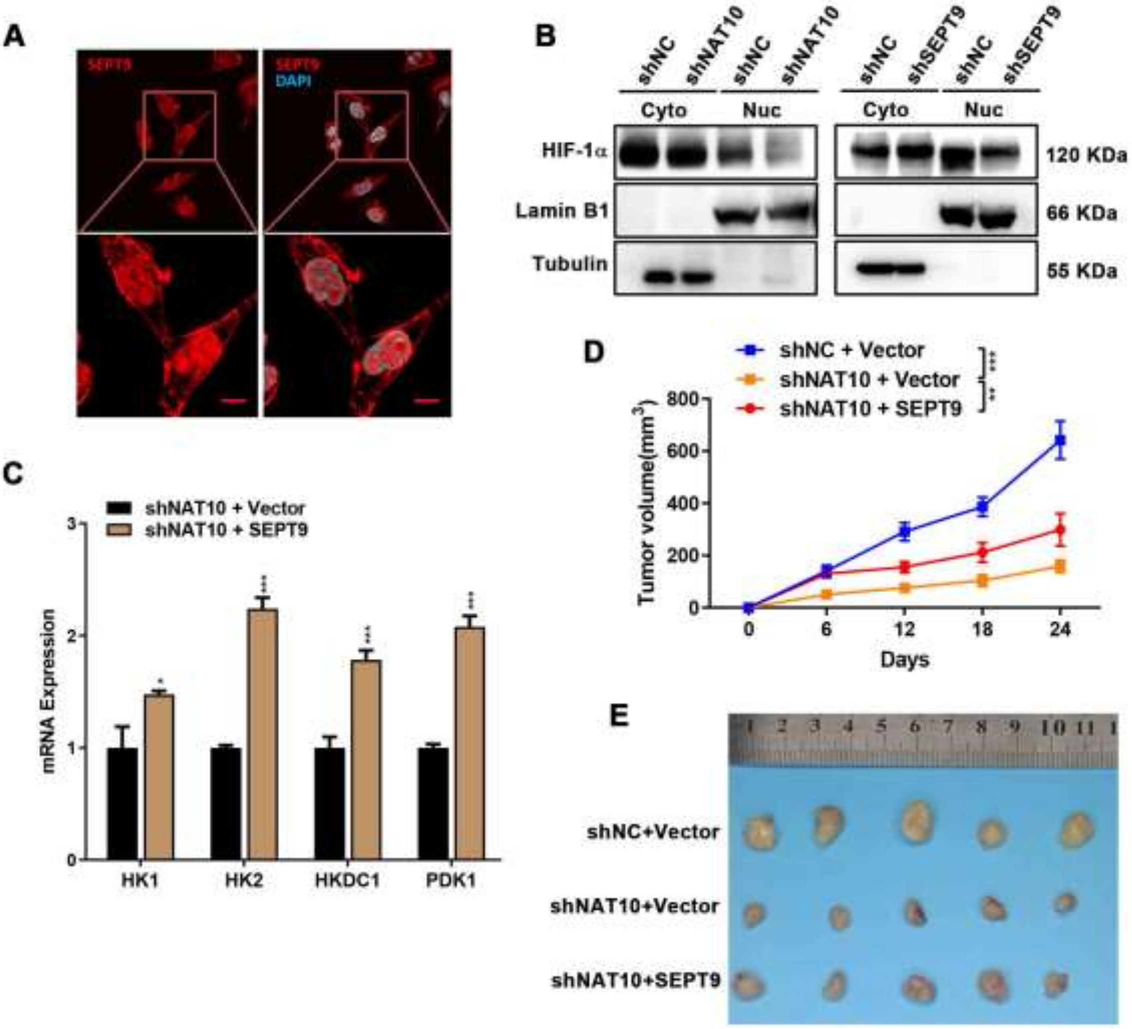

Figure S5. SEPT9 promotes the translocation of HIF-1α into the nucleus.

(A) MGC 80-3 cells were grown and subjected to immunofluorescence staining (SEPT9, red; DAPI, blue) and confocal microscopy. Scale bars, 10 μm. (B) Reduced HIF-1α was observed in the nucleus of AGS cells with NAT10 or SEPT9 knockdown. (C) Expression of HK1, HK2, HKDC1 and PDK1 was restored in shNAT10+SEPT9 AGS cells compared to the control cells. (D) Tumor growth curve of different MGC 80-3 cells in the xenograft mouse model was based on tumor size measurement. (E) Tumor images of xenograft nude mice model subcutaneously injected with MGC 80-3 cells.

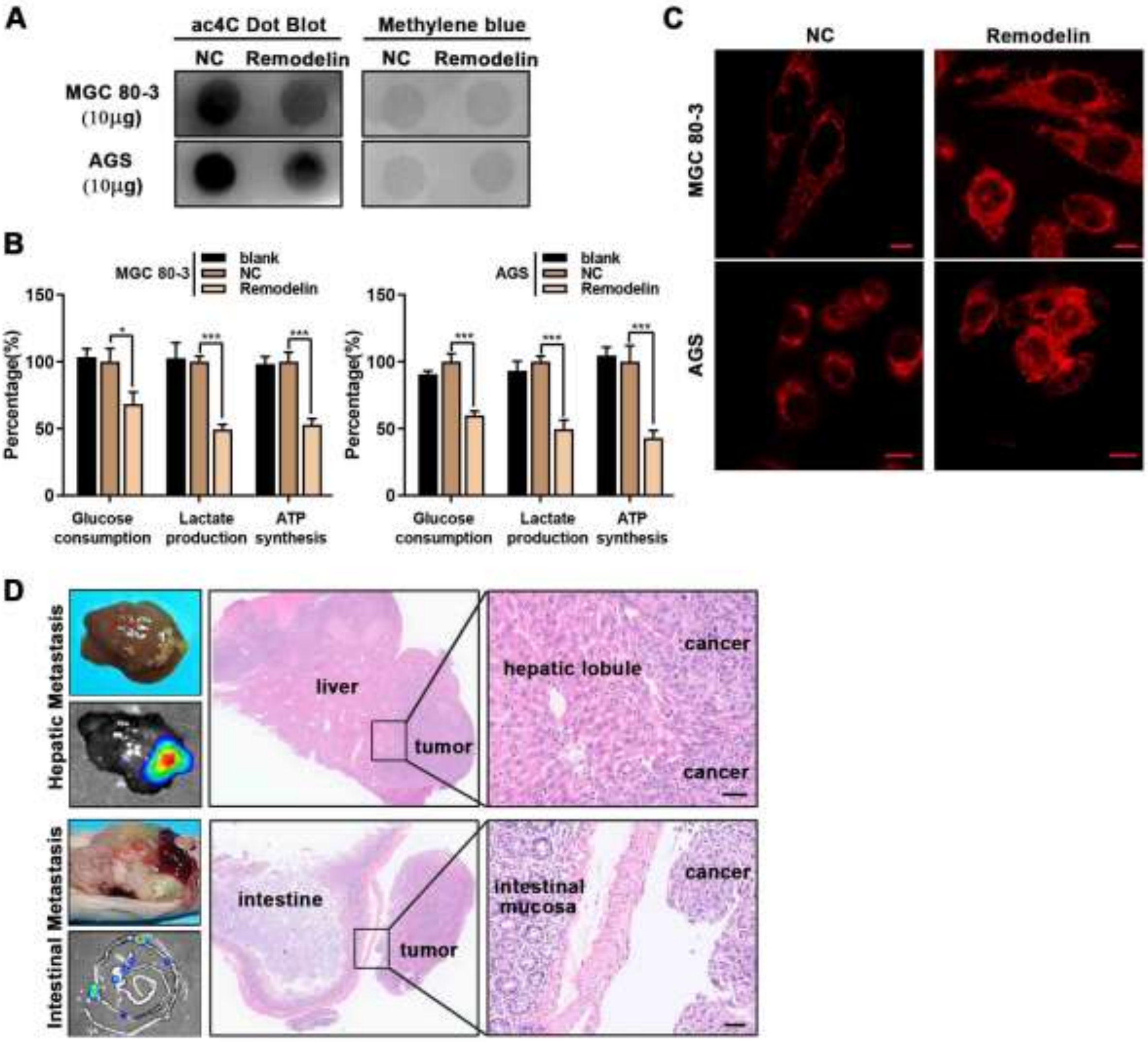

Figure S6. Remodelin effectively reverses the glucose metabolic reprogramming in GC cells.

(A) The total RNA ac4C level was decreased in Remodelin (25 µM) treated MGC80-3 and AGS cells. (B and C) Remodelin treated MGC80-3 and AGS cells exhibited lower levels of glycolysis (B) and less mitochondrial damage (C). The GC cells were treated with 100 µM $CoCl_2$. Scale bars, 10 µm. (D) Representative images of metastatic nodules (indicated by red circles or fluorescence) were shown and detected by hematoxylin-eosin (H&E) staining. Scale bars, 50 µm.

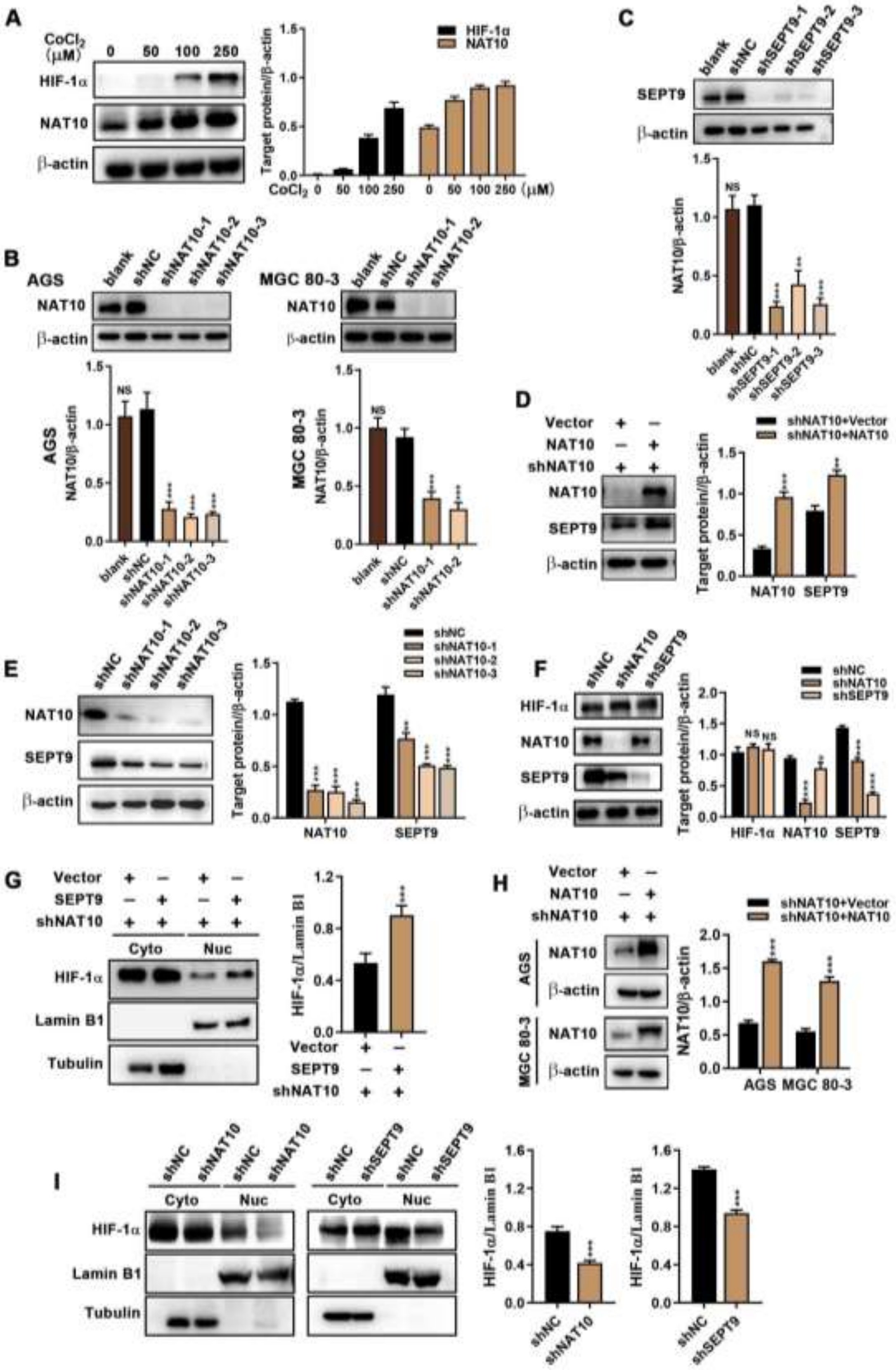

Figure S7. The corresponding quantitative analysis of western blot results.

(A) Western blot analysis of NAT10 expression in AGS cells treated with 0-250 µM $CoCl_2$. (B) Expression of NAT10 in shRNAs transfected AGS (left) and MGC 80-3 (right) cells was analyzed by Western blot and qPCR. (C) Expression of SEPT9 in shRNAs transfected AGS

cells were analyzed by Western blot and qPCR. (D and E) NAT10 promoted the protein expression of SEPT9. (F) Western blot analysis of the expression of HIF-1α in shNAT10 or shSEPT9 AGS cells. (G) shNAT10+SEPT9 AGS cells were subjected to nuclear and cytoplasmic proteins extraction. (H) Expression of NAT10 in shNAT10+NAT10 AGS and MGC 80-3 cells, as measured by Western blot. (I) Reduced HIF-1α was observed in the nucleus of AGS cells with NAT10 or SEPT9 knockdown.

Supplementary tables

Table S1. The plasmids used in this study

Plasmids		Catalog number	Source
Vector	Vector-EGFP (GV230)	CON083	Genechem
shRNA plasmids	shNAT10 1-3	HSH105950-LVRU6GP	GeneCopoeia
	shSEPT9 1-3	CS-HSH057040-LVRU6GP-01	
	shNC	CSHCTR001-LVRU6GP	
Overexpression (OE) plasmid	Vector	EX-NEG-Lv201	GeneCopoeia
	NAT10	EX-I5674-Lv201	
	SEPT9	EX-H3579-Lv206	
Luciferase reporter plasmids	SEPT9 3’UTR-WT	CS-HmiT070972-MT06-01	GeneCopoeia
	SEPT9 3’UTR-Mut	CS-HmiT070972-MT06-02	
	NAT10-promoter-WT	CS-HPRM35895-PL01-01	
	NAT10-promoter-Mut	CS-HPRM35895-PL01-02	

Table S2. The primer sequences used for qPCR in this study

Genes	Primers sequences
NAT10	5'-ATAGCAGCCACAAACATTCGC-3'
	5'-ACACACATGCCGAAGGTATTG-3'
SEPT9	5'-GCAGAGCGGCTTGGGTAAAT-3'
	5'-ATATCGTGCGTGATGGACTTG-3'
HIF-1α	5'-GAACGTCGAAAAGAAAAGTCTCG-3'
	5'-CCTTATCAAGATGCGAACTCACA-3'
β-actin	5'- TTGTTACAGGAAGTCCCTTGCC -3'
	5'- ATGCTATCACCTCCCCTGTGTG -3'
HK1	5'-GCTCTCCGATGAAACTCTCATAG-3'
	5'-GGACCTTACGAATGTTGGCAA-3'
HK2	5'-CCCAGCACAAAGCAGTC-3'
	5'-TCCGTGAAGAAGTAGGCAA-3'
HKDC1	5'-GTTGCCCACCTTCGTCAGG-3'
	5'-AGCGACTTGCACCTTCAGC-3'
PDK1	5'-GAGAGCCACTATGGAACACCA-3'
	5'-GGAGGTCTCAACACGAGGT-3'

Table S3. The antibodies used in this study

Primary antibodies	Catalog number	Source
Anti-NAT10 (1:1000)	13365-1-AP	proteintech
Anti-N4-acetylcytidine(ac4C) (1:1000)	ab252215	Abcam
Anti-β-actin (1:1000)	ab8226	Abcam
Anti-SEPT9 (1:1000)	10769-1-AP	proteintech
Anti-HIF-1α (1:500)	H1alpha67	Novus
Anti-CD31 (1:1500)	GB113151	Servicebio
DAPI	#4083 MSDS	Cell Signaling Technology
Anti-Mouse IgG H&L (HRP)	Ab136815	Abcam
Anti-Rabbit IgG H&L (HRP)	Ab136817	Abcam
Anti-Rabbit IgG (H+L), F(ab')2 Fragment (Alexa Fluor® 555 Conjugate) (red) (1:1000)	#4413	Cell Signaling Technology
Anti-Mouse IgG (H+L), F(ab')2 Fragment (Alexa Fluor® 488 Conjugate) (green) (1:1000)	#4408	Cell Signaling Technology

Table S4. The critical commercial assays used in this study

Reagents	Catalog number	Source
Lenti-Pac HIV Expression Packaging Kit	LT002	Genecopoeia
TB Green Premix Ex Taq II Kit	RR820A	Takara
Glucose Assay Kit	MAK263	Sigma-Aldrich
Lactate Assay Kit	MAK064	Sigma-Aldrich
Enhanced ATP assay Kit	S0027	Beyotime Biotechnology
Duo-Luciferase HS Assay Kit	LF004	GeneCopoeia
RIP Kit	Bes5101	BersinBio
Epi™ ac4C immunoprecipitation kit	R1815	Epibiotek
protein A/G magnetic beads	8880210002D/10004D	Invitrogen
Epi™ Ribosome Profiling Kit	R1814	Epibiotek
RNA Clean&Concentrator-5 kit	R1016	ZYMO
EpiTM RiboRNA Depletion Kit	R1805	Epibiotek
VAHTS Stranded mRNA-seq Library Prep Kit	NR612-02	Vazyme Biotech
GL DNA Marker 1000	AG11907	Accurate Biotechnology

Table S5. Results of molecular docking

Receptor	Ligand	Binding energy	Interface area (Å²)	Hydrogen bonds (SEPT9: HIF-1α)
SEPT9	HIF-1α	-9.7 kcal/mol	1234.1	GLY 78: PHE 295<br>VAL 79: THR 296<br>ASN 252: ALA 281<br>THR 254: TYR 276<br>TYR 260: SER 274

	ARG 262: THR 288
	ASP 206: ASN 326
	ASP 209: ASN 326
	GLU 205: ASN 329
